# In Silico Identification of the Complex Interplay between Regulatory SNPs, Transcription Factors, and Their Related Genes in *Brassica napus* L. Using Multi-Omics Data

**DOI:** 10.3390/ijms22020789

**Published:** 2021-01-14

**Authors:** Selina Klees, Thomas Martin Lange, Hendrik Bertram, Abirami Rajavel, Johanna-Sophie Schlüter, Kun Lu, Armin Otto Schmitt, Mehmet Gültas

**Affiliations:** 1 Breeding Informatics Group, Department of Animal Sciences, Georg-August University, Margarethe von Wrangell-Weg 7, 37075 Göttingen, Germany; selina.klees@uni-goettingen.de (S.K.); thomasmartin.lange@stud.uni-goettingen.de (T.M.L.); hendrik.bertram@stud.uni-goettingen.de (H.B.); abirami.rajavel@uni-goettingen.de (A.R.); j.schlueter01@stud.uni-goettingen.de (J.-S.S.); armin.schmitt@uni-goettingen.de (A.O.S.); 2 College of Agronomy and Biotechnology, Southwest University, Chongqing 400715, China; drlukun@swu.edu.cn; 3 Academy of Agricultural Sciences, Southwest University, Chongqing 400715, China; 4 State Cultivation Base of Crop Stress Biology for Southern Mountainous Land of Southwest University, Chongqing 400715, China; 5 Center for Integrated Breeding Research (CiBreed), Albrecht-Thaer-Weg 3, Georg-August University, 37075 Göttingen, Germany

**Keywords:** rSNPs, transcription factor, multi-omics, gene expression, random forest, DOF

## Abstract

Regulatory SNPs (rSNPs) are a special class of SNPs which have a high potential to affect the phenotype due to their impact on DNA-binding of transcription factors (TFs). Thus, the knowledge about such rSNPs and TFs could provide essential information regarding different genetic programs, such as tissue development or environmental stress responses. In this study, we use a multi-omics approach by combining genomics, transcriptomics, and proteomics data of two different *Brassica napus* L. cultivars, namely Zhongshuang11 (ZS11) and Zhongyou821 (ZY821), with high and low oil content, respectively, to monitor the regulatory interplay between rSNPs, TFs and their corresponding genes in the tissues flower, leaf, stem, and root. By predicting the effect of rSNPs on TF-binding and by measuring their association with the cultivars, we identified a total of 41,117 rSNPs, of which 1141 are significantly associated with oil content. We revealed several enriched members of the TF families DOF, MYB, NAC, or TCP, which are important for directing transcriptional programs regulating differential expression of genes within the tissues. In this work, we provide the first genome-wide collection of rSNPs for *B. napus* and their impact on the regulation of gene expression in vegetative and floral tissues, which will be highly valuable for future studies on rSNPs and gene regulation.

## 1. Introduction

With rapidly evolving genomic sequencing technologies, the number of identified single nucleotide polymorphisms (SNPs) is increasing at a remarkable pace. Due to their straightforward functional interpretation, SNPs located in the protein coding regions of the genes are mostly in the focus of research. However, results from genome-wide association studies (GWAS) reveal that the vast majority of phenotype-associated SNPs are located in intergenic and intronic regions [1,2]. Many of these non-coding SNPs are located within the regulatory regions, such as the promoter regions, and could hence influence the gene expression by changing the binding affinity of regulatory proteins. In recent years, these so-called regulatory SNPs (rSNPs) have come into the focus of research and the underlying mechanisms resulting in a differential gene expression are closely studied for many specific traits and diseases [2,3]. It is well known that the differential gene expression in different tissues and under certain environmental conditions is governed by the binding of transcription factors (TFs) to specific DNA-sequence motifs, the transcription factor binding sites (TFBSs). By altering the sequence within such a TFBS, an rSNP can have a severe effect on TF binding and, hence, could change a gene’s expression rate [2,3,4]. In plant sciences, previous studies identified different putative rSNPs affecting different traits, as e.g., seed shattering in rice [5], maize rough dwarf disease [4], grain weight in wheat [6], or vicine and convicine content of *Vicia faba* [7]. Until now, there are several tools predicting a SNP’s impact on TF binding (e.g., [3,8,9,10,11,12]), but the Regulatory Sequence Analysis Tool (RSAT) [11] is one of the few tools supporting plants. In RSAT, users have the possibility to retrieve specific genetic variants with the corresponding flanking sequences and predict their impact on TF binding in a variety of organisms [11]. However, all these studies and tools concentrate on single regulatory variants and do not cover a systematic analysis to obtain a genome-wide prediction of rSNPs. Notwithstanding that the importance of rSNPs and their regulatory power is well known, no such systematic analysis including a genome-wide prediction of rSNPs for *Brassica napus* L. exists.

As an important oilseed crop, *B. napus* is grown and used worldwide for its oil and fodder production where the oil is widely used for human consumption and biofuel production, while the rapeseed meal remaining after oil extraction can be used as high-protein animal fodder [13,14]. *B. napus* has gained global importance due to an intensive breeding program focusing on the reduction of nutritionally undesirable components in the oil and fodder and thus, enabled the production of varieties with both low erucic acid and glucosinolate content [15]. Today, improving the oil content is an important breeding goal and in this context the resistance to several stresses is a relevant objective [13,16,17]. The oil is stored within the seeds as triacylglycerols (TAGs) in oil bodies, but the TAG synthesis takes place in the plastids through a variety of different interacting metabolic pathways and regulatory processes [18]. However, such pathways as well as the underlying transcriptional machinery controlling the oil content and -quality could vary across different *B. napus* cultivars [19,20]. Hence, the investigation of such biological processes is an important task to assess the genetic programs of two cultivars: (i) Zhongshuang11 (ZS11) characterized by a double-low accession (00, low erucic acid and low glucosinolate) and a high oil content and; (ii) Zhongyou821 (ZY821) with double-high accession (++, high erucic acid and high glucosinolate) and low oil content [19].

To unravel such genetic programs in both *B. napus* cultivars, we computationally identified the regulatory processes controlling specific biological functions associated with oil content, plant growth, or responses to environmental stresses. For this purpose, we used multi-omics data including genomics and transcriptomics data of two cultivars and plant proteomics data to identify rSNPs, important genes and transcriptional regulators orchestrating specific genetic programs in different tissues and thus, leading to phenotypic differences of both cultivars. To this end, mainly focusing on the vegetative and floral tissues such as flower, leaf, stem, and root, we first identified differentially expressed genes (DEGs) between both cultivars in these four tissues. Second, by analyzing 670,028 high-quality SNPs, we obtained a genome-wide collection of rSNPs and their predicted consequences on the binding affinity of the TFs. Similar to our previous studies [21,22], we applied a random forest (RF) feature selection approach to assess the importance of rSNPs with respect to the phenotype. Subsequently, we determined tissue-specific DEGs harboring those important rSNPs within their promoter region, whose transcription is likely to be affected by the consequences of rSNPs on TF binding. By causing a disruption of a TFBS or the creation of a new TFBS, rSNPs can strongly influence the binding affinity of TFs and, thus, can lead to the differentiation of a wide range of genetic processes in both cultivars like their oil content, tissue development, or stress-resistance mechanisms [13,23]. Our results show that the consideration and systematic analysis of multi-omics data (genomics, transcriptomics, and proteomics) of two different *B. napus* cultivars provides: (i) essential information about functions of transcription factors involved in the regulation of transcriptional activity of vegetative and floral tissues; and (ii) novel insights into the regulatory programs controlling oil content and -quality underlying both cultivars.

## 2. Results and Discussion

### 2.1. Differentially Expressed Genes

The comparison of the ZS11 (high oil content, double-low cultivar) against the ZY821 (low oil content, double-high cultivar) in the four tissues revealed several differentially expressed genes, of which the up-regulated DEGs refer to genes with a significantly higher expression in ZS11 than in ZY821, whereas down-regulated genes are significantly higher expressed in ZY821 than in ZS11 (Table 1, the full lists of DEGs is given in Table S1). The overlap of the four tissues showed that 171 and 252 DEGs were found up- and down-regulated in all four tissues, respectively. To assess the underlying biological processes, we provide the Gene Ontology (GO) terms and treemaps for the respective up- and down-regulated DEG sets in Table S2 and Figure S1.

### 2.2. Transcription Factor Binding Site Enrichment Analysis

**Figure 1:**
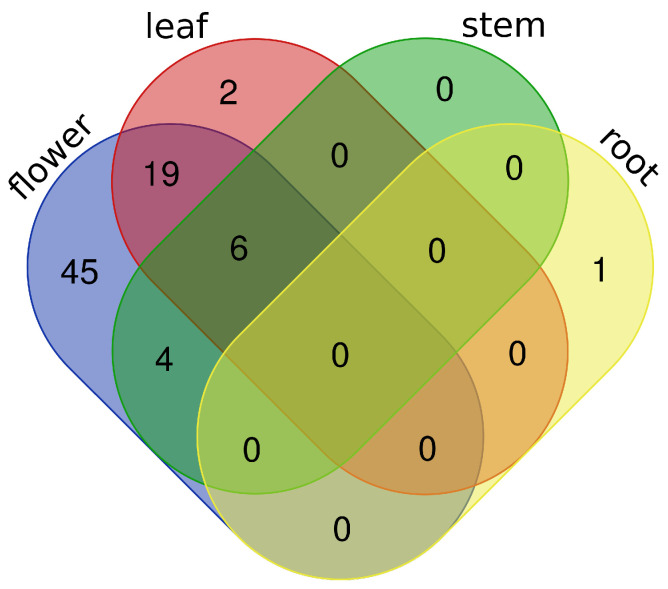
Venn diagram for the enriched transcription factors (TFs) found for the tissues flower, leaf, stem, and root of *B. napus* (visualized with http://bioinformatics.psb.ugent.be/webtools/Venn/).

For understanding the expression behavior of DEGs regarding their up- or down-regulation, the knowledge on the TFs, which are involved in controlling the regulatory programs of these genes, is important to explain gene expression changes between both *B. napus* cultivars. Applying TFBS enrichment analyses, we assessed the potential roles of TFs in the regulation of the DEGs based on the over-representation of their TFBSs in the promoter regions. In the following, we refer to a TF as enriched in a tissue, if its respective TFBS is significantly over-represented in the set of promoter sequences of the DEGs in that tissue. The results of these analyses show that the number of enriched TFs is remarkably different between tissues: While the largest number of enriched TFs was identified in the flower tissue (74), there was only one enriched TF in the root tissue. We further found 27 and 10 TFs enriched in the leaf and stem tissues, respectively (Figure 1; the complete list of enriched TFs is given in Table S3).

Interestingly, Figure 1 shows that the number of unique enriched TFs found for flower is clearly higher than those of the remaining tissues. In this regard, the transcription factor GATA19 found only for the root tissue is a member of GATA-type zinc finger proteins, which are known to be involved in light-mediated gene expression and nitrogen-dependent stress response [24,25].

Furthermore, the TCP family members TCP16 and ARALYDRAFT_897773 (also known as TCP4) were identified as enriched only in the leaf tissue. As shown in previous studies, TCP genes participate in the developmental control of plant form as, e.g., flower and leaf shape or shoot branching by regulating cell proliferation and they have been shown to be highly expressed in leaf [26,27].

Moreover, a minority of the TFs (PIF1, PIF7, bHLH74, UNE10, OJ1058_F05.8, and BEH2) are simultaneously enriched for flower, leaf, and stem tissues. Besides the two phytochrome interacting factors (PIF1 and PIF7), the factors PIF3, PIF4, and PIF5 are enriched only in flower and leaf. The PIFs belong to one of the largest classes of plant TFs, the basic/helix-loop-helix (bHLH) proteins [20], and they are known to repress photomorphogenesis in darkness by promoting the transcription of genes which positively regulate cell elongation in *A. thaliana* [28]. In particular, while PIF1 has been reported to negatively regulate seed germination in response to light and hormone signaling [29,30], PIF4 and PIF5 are regulators of de-etiolation [31], and PIF7 is a main regulator of stem elongation in light [32].

The factor unfertilized embryo sac10 (UNE10) is another member of the bHLH class. It is supposed to inhibit far-red light signaling by interacting with phytochromes [33] and to play an important role during the fertilization of ovules by pollen in *A. thaliana* [34].

Several BES1 (BRI1-EMS-SUPPRESSOR1) family members, in particular BEH2, BEH3, BEH4, and BZR1, are enriched in flower and leaf and/or stem tissues, and are known to regulate brassinosteroid-mediated genes. Different BES1-family members are suggested to regulate different auxin and jasmonic acid-related genes, resulting in enhanced growth and vigor [35,36] in *B. napus* and *A. thaliana* and to be involved in stress resistance such as salt and drought stress in *B. napus* and *B. rapa* [37,38].

Interestingly, we found members of the TF families MYB (or MYB-related; MYB46, MYB98, MYB119, MYB59, and MYB111), DOF type C2H2 zinc finger factors (DOF4.2, OBP3, AT2G28810 (DOF2.2), AT5G02460 (DOF5.1), and AT5G66940 (DOF5.8)), and NAC (NAC080, NAC028, NAC025, NAC058, NAC055, NAC043, NAC083, and T11I18.17) enriched exclusively in flower. The MYB TFs are involved in several processes as, e.g., response to biotic and abiotic stress, development, and differentiation; in particular, MYB46 is involved in secondary wall and fiber biosynthesis; MYB98 and MYB119 are important regulators of female gametophyte development; MYB59 is involved in cell cycle progression, and MYB111 plays a crucial role in flavonol biosynthesis in *A. thaliana* [39,40,41,42].

On the other hand, the DOF proteins are characterized by a highly conserved DNA binding domain (DOF domain) and are present in many different plant species [43]. DOF proteins play a role in several biological processes as, e.g., flowering time, seed development, and responses to hormones and abiotic stress [43,44,45]. Interestingly, He et al. [44] found the *Arabidopsis* DOF5.8 to be an upstream regulator of a gene encoding an NAC family member in response to drought and salt stress.

The NAC transcription factors make up one of the largest plant-specific TF families with specific functions regarding plant development, biotic stress response, and response to environmental stress [46]. Research performed in *B. napus* revealed upregulation of NAC genes after mechanical wounding and infection with *Sclerotinia sclerotiorum*. In the same way, NAC genes were upregulated after the induction of a cold shock [47]. Interestingly, we have shown that members of TF families such as GATA, DOF, NAC, or MYB are important regulators of genes with a monotonic expression pattern in both cultivars in the seed tissue by forming TF co-operations [48].

### 2.3. Analysis of Regulatory SNPs

**Figure 2:**
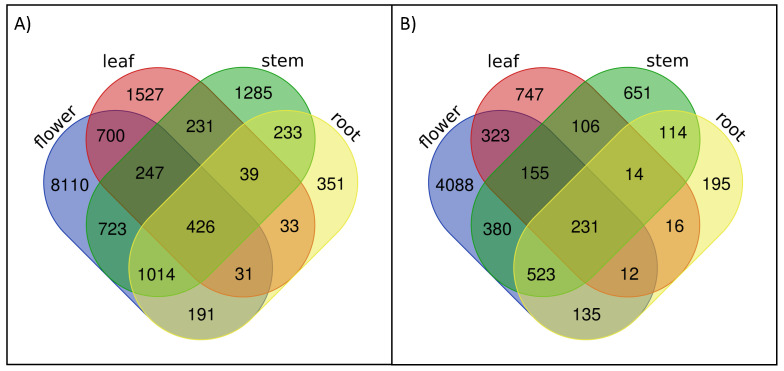
Overlap of the DEGs in (**A**) and rSNPs in (**B**) for the four investigated tissues (visualized with http://bioinformatics.psb.ugent.be/webtools/Venn/).

Today it is well known that the binding affinity of TFs can be affected by rSNPs to a great extent and, hence, either enable or repress the protein–DNA interaction. In order to be able to explain the observed differences in the expression of the DEGs, we investigated the role of rSNPs causing such severe effects on TF binding. Taking the initial 670,028 high-quality SNPs into account, we determined 41,117 of them as rSNPs due to their genomic positions in the promoter regions of *B. napus* genes and their consequences of either “Gain of TFBS” or “Loss of TFBS”. A closer look at these rSNPs reveals that 5847 (flower), 1604 (leaf), 2174 (stem) and 1240 (root) rSNPs are related to the DEGs (the full list of rSNP predictions can be found in Table S4). Interestingly, a direct comparison of the rSNP and DEG numbers shows that approximately 50% of DEGs contain on average one rSNP within the promoter region (Figure 2).

**Figure 3:**
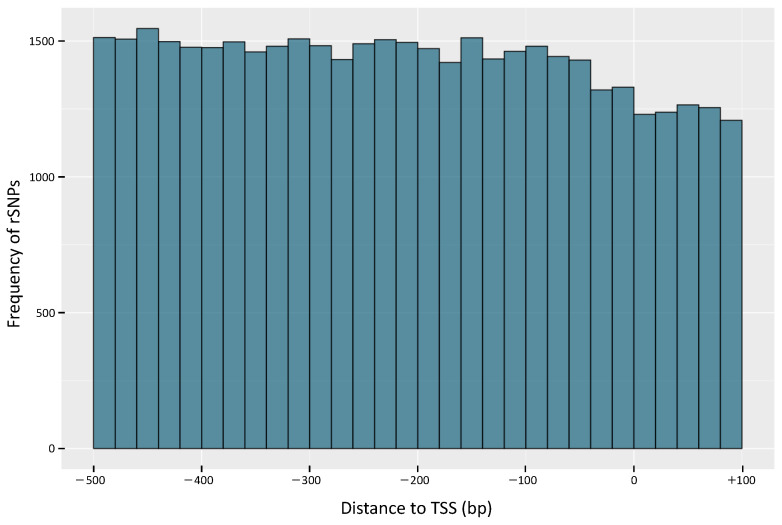
Distribution of rSNPs relative to the transcription start sites (TSS) of the corresponding genes. Position 0 denotes the position of the TSS.

To gain a better insight into the distribution of the rSNPs in the promoter regions, we investigated their genomic positions relative to the transcription start sites (TSS). The results of this analysis indicate that while there are fewer rSNPs around the TSS, we observed a tendency of increasing rSNP numbers in the remaining upstream promoter regions (Figure 3). This finding goes in line with the observation of Triska et al. [49], who performed a similar analysis based on the SNP distributions in the promoters of rice.

### 2.4. Analysis of Important Regulatory SNPs

**Figure 4:**
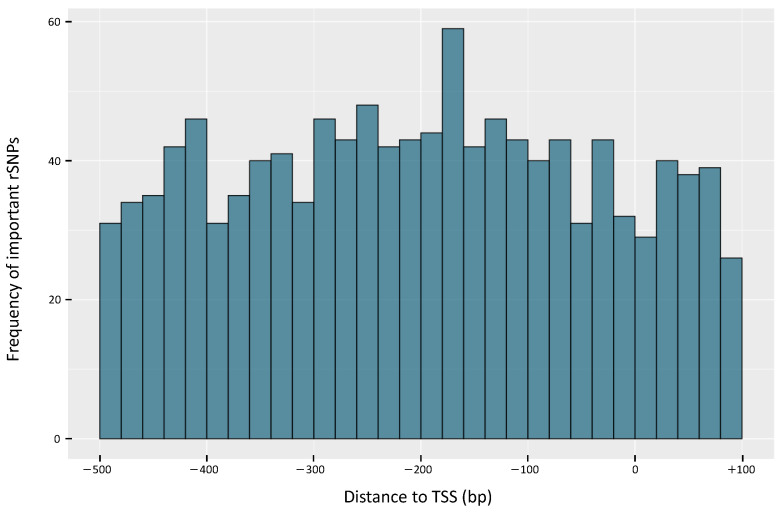
Distribution of *important rSNPs* relative to the transcription start sites (TSS) of the corresponding genes. Position 0 denotes the position of the TSS.

Moreover, we assessed the importance of rSNPs regarding their significant association with oil content of both cultivars and identified 1141 *important rSNPs* (the complete list of *important rSNPs* is given in Table S5). The consideration of the *important rSNPs* in the DEGs of the tissues consequently results in 245 *important rSNPs* in the flower, 68 in the leaf, 142 in the stem and 82 in the root tissue. Surprisingly, the distribution of *important rSNPs* relative to the TSS (see Figure 4) shows a behavior in the promoter regions, that is remarkably different from that of rSNPs (Figure 3). This finding suggests that the *important rSNPs* do not follow a certain pattern but rather spread throughout the considered promoter regions.

### 2.5. DEGs Harboring Important rSNPs in the Promoter Region

In order to assess the regulatory impact of *important rSNPs* on the regulation of the DEGs and, hence, to explain their differential expression status, we identified the regulatory interplay between rSNPs, TFs and their corresponding DEGs of interest.

As a result, we found 145, 44, 81, and 50 DEGs harboring *important rSNPs* in the promoter region for flower, leaf, stem, and root, respectively.

To gain a deeper insight into the functions of these genes, we identified their related enriched GO terms as well as the pathways (Table S6). Regarding enriched GO terms of biological processes, we could observe DEGs related to protein folding, alcohol, lipid or phytosteroid biosynthesis in leaf and a variety of genes related to oxidation–reduction processes in the leaf and flower tissue.

Interestingly, the gene *BnaA06g33360D* occurs in the flower and stem set of DEGs harboring *important rSNPs*, which leads to the significant enrichment of the monoterpenoid biosynthesis pathway [50]. Monoterpenoids are volatile secondary plant products that could play a role in olfactory cues for pollinating insects in *A. thaliana* [51]. Surprisingly, the gene *BnaA06g33360D*, which presumably codes for a monoterpene synthase, is down-regulated in flower tissue, while it is up-regulated in stem tissue.

In the gene set of the leaf tissue, several KEGG pathways [50] related to fatty acid metabolism were enriched. Especially the gene *BnaA04g26960D*, which is significantly up-regulated in the leaf tissue, is represented in the enriched pathways fatty acid metabolism, fatty acid biosynthesis, fatty acid degradation or peroxisome. *BnaA04g26960D*, also called *BnaLACS1-4*, is a member of the long-chain Acyl-CoA synthetase (*LACS*) family of genes, which have been shown to be involved in fatty acid biosynthesis in chloroplasts and seed oil accumulation in *B. napus* [52]. Furthermore, several *LACS* genes showed differential gene expression in multiple tissues in the comparison between high and low oil content *B. napus* cultivars [52]. Within the promoter region of *BnaA04g26960D*, we identified one *important rSNP* (chromosome A04, position 19042835, C → T) which causes a “Gain of TFBS” for the binding site of MNB1A (the maize DOF1 TF) 90 bp downstream of the TSS. More specifically, this means the DOF1 binding site is not present in the reference allele (C), while DOF1 binding is likely to be enabled by the alternate allele (T). The importance of DOF-mediated gene regulation has already been shown in the results of TF enrichment (see Section 2.2). Interestingly, the soybean DOF proteins GmDOF4 and GmDOF11 have been shown to directly induce *LACS* genes, and also increased the fatty acid content in transgenic *Arabidopsis* seeds [43,53]. In cotton, an overexpression of the *GhDOF1* gene led to an increase of lipid levels in the seeds [43,54]. These results suggest that this *important rSNP* might play an important role in the DOF1-mediated expression rate of the *LACS* gene *BnaA04g26960D* and, hence, might regulate the fatty acid content in *B. napus*.

The gene *BnaC08g26140D*, present in the significantly enriched pathways fatty acid metabolism, biosynthesis of unsaturated fatty acids and fatty acid elongation of the leaf gene set, encodes a Trans-2,3-enoyl-CoA reductase (ECR). This enzyme is involved in the synthesis of very-long-chain fatty acids (VLCFAs) which are essential for the synthesis of cuticular waxes, sphingolipids and Triacylglycerols (TAGs) in *B. napus* [55]. As an enzyme of VLCFA synthesis, it is also known to catalyze the fourth reaction of the elongase complex during erucic acid synthesis [55,56]. Surprisingly, we found the *ECR* gene up-regulated in the double-low cultivar with high oil content. One possible explanation for its up-regulation in the low erucic acid cultivar might be that the synthesized VLCFAs are precursors for a variety of different lipids in higher plants, such as cuticular waxes [55].

In the promoter region of the *B. napus ECR* gene, we found three *important rSNPs* (hereinafter referred to as ECR-rSNP1, ECR-rSNP2 and ECR-rSNP3), affecting five different binding sites. ECR-rSNP1 (chromosome C08, position 27619847, G → T) is positioned −152 bp from the TSS and causes a “Loss of TFBS” for the *Arabidopsis* response regulator (ARR10) or response regulator 10 (RR10) binding site. As a cytokin response regulator, RR10 is involved in cytokinin-mediated signaling pathways and acts, e.g., as negative regulator of drought response in *A. thaliana* [57]. In *B. napus*, it has been shown to be up-regulated in leaves under salt stress [58]. The ECR-rSNP2 (chromosome C08, position 27619942, 247 bp upstream of the TSS, A → G) causes a “Loss of TFBS” for TF DOF4.5 and a “Gain of TFBS” for TF MYB56. The DOF4.5 is another member of the DOF family of TFs, which is assumed to share regulatory functions in, e.g., shoot branching and seed coat formation together with other DOF family members in *A. thaliana* [45]. MYB56 is a member of the previously described MYB family and is known to be a positive regulator of seed size and to control seed coat development in *Arabidopsis* [18,59]. The ECR-rSNP3 (258 bp upstream of the TSS) causes a “Loss of TFBS” for TF DOF4.5 and a “Gain of TFBS” for ethylene-responsive transcription factor ERF069. Within the AP2/EREBP superfamily of TFs, ERF069 belongs to the ethylene-responsive element binding proteins (EREBP) subfamily, which are known to respond to abiotic stress [60]. Liu et al. observed an up-regulation of ERF069 in response to chromium treatment in *A. thaliana* [60]. In the foxtail millet, *SiAP2/ERF-069* was up-regulated under drought and salinity stress [61] and in *B. napus* and *ERF069* was up-regulated under Pi-starvation in 3- and 5-leaf stage seedings [62].

With this analysis, we identified several interesting tissue-specific DEGs whose regulation is likely to be influenced by the “Loss-” or “Gain of a TFBS” caused by an *important rSNP* within their regulatory region. The TFs overlapping these *important rSNPs* provide a promising basis for further investigation of their regulatory roles and underlying pathways that lead to the distinction between the two cultivars.

## 3. Materials and Methods

**Figure 5:**
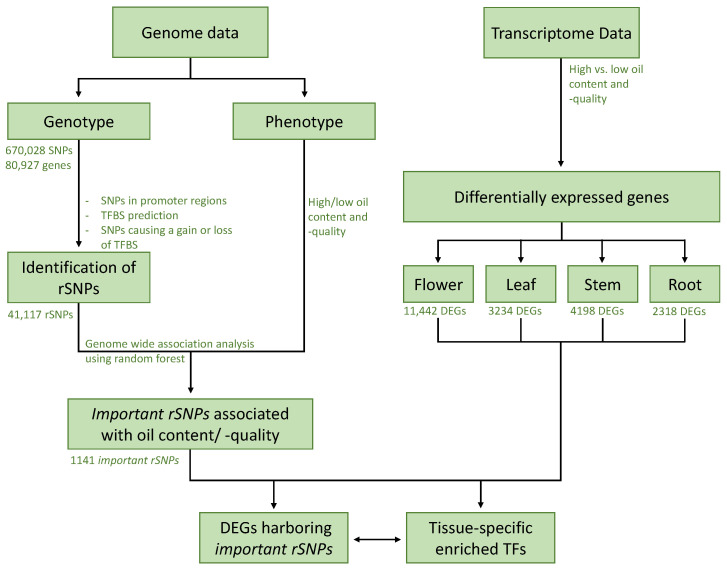
Flowchart of the analysis applied in this study.

Our analysis framework follows the structure shown in Figure 5, i.e., we start with the analysis of genomics and transcriptomics data to systemically monitor the important (tissue-specific) regulatory SNPs and TFs regulating the DEGs.

### 3.1. B. napus Data Set and Data Preparation

In this study, we use publicly available genomics and transcriptomics data sets of two *B. napus* cultivars, which are briefly explained below. Readers who are interested in learning more about these data sets are kindly referred to the original study [19].

#### 3.1.1. Genotype Data

To identify the rSNPs that are likely to be associated with different genetic programs in two *B. napus* cultivars, namely Zhongshuang11 (ZS11) with double-low accession (low erucic acid and glucosinolate, 00) and high oil content and Zhongyou821 (ZY821) with double-high accession (high erucic acid and glucosinolate, ++) and low oil content, we analyzed a genotype data set that has previously been used in [19]. Prof. Kun Lu from the Southwest University, China provided the genotype data set for this study. The raw sequencing data are available at the BIG Data Center under BioProject accession code PRJCA000376. The genotype data set comprises 670,028 high-quality SNPs (MAF > 0.05) for 280 Zhongshuang11 (ZS11) and 133 Zhongyou821 (ZY821) samples. The data set contains SNPs which are located on the chromosomes A01-A10 and C01-C09 (originated during hybridization of *B. rapa* (AA, 2n = 20) and *B. oleracea* (CC, 2n = 18) [19]) including 80,927 genes.

#### 3.1.2. Transcriptome Data

The RNA-sequencing data of four tissues (flower, leaf, stem, and root) from both cultivars (ZS11 and ZY821) with two biological replicates each were generated by Lu et al. [19]. The raw sequencing data were downloaded from the BIG Data Center under BioProject accession code PRJCA001246. In line with [19], we mapped the filtered reads to the *B. napus* reference genome version 4.1 (obtained from [63] and available at https://wwwdev.genoscope.cns.fr/brassicanapus/data/) using STAR 2.4.2a [64]. Finally, applying the htseq-count program [65] to the aligned sequencing reads, we identified the number of reads (gene count table).

For the identification of differentially expressed genes (DEGs), we applied the DESeq2 tool (R package version 1.24.0) with default settings in the median-of-ratios normalization method, fold change shrinkage and a significance cutoff of an absolute log${}_{2}$ fold change of 2 and an adjusted *p*-value of 0.05 [66]. The experimental design of the differential expression analysis is shown in Table 2.

### 3.2. Transcription Factor Binding Site Enrichment Analysis in Promoter Sequences

In order to identify transcription factors (TFs) with significantly over-represented transcription factor binding sites (TFBSs) in the promoter sequences of the DEGs, we employed the CiiiDER algorithm [67].

However, the selection of the promoter regions is crucial: (i) to avoid the redundancy between sequences which could lead to the overestimation of some TFBSs [68] (ii) to address the inaccuracy of transcription start site (TSS) positions resulting from their imprecise prediction. To overcome these issues, we followed a similar strategy to those suggested in previous studies [7,49,68,69,70,71,72,73,74] and accordingly extracted two sets of promoter sequences for each tissue ranging from −500 bp to +100 bp relative to the TSS using the reference genome version 4.1 and gene annotation given in [63]. While the first sequence set refers to the promoter sequences of the DEGs (foreground set), the second set contains the promoter sequences of genes having the same GC-content as the foreground set (background set) [75]. For the generation of background sets, we used the oPOSSUM3.0 [76] web application (http://opossum.cisreg.ca/GC_compo/) and selected only sequences that are not included in the foreground set. Second, following the workflow of the CiiiDER program [67], we scanned each sequence by applying the MATCH™ program [77] with a non-redundant plant position weight matrix (PWM) library from the JASPAR database [78] to detect the potential TFBSs. Finally, comparing the distribution of TFBSs predicted in the foreground as well as the background promoter sequence set, the enrichment of TFBSs was assessed (Bonferroni adjusted *p*-value threshold of 0.01).

### 3.3. Identification of Regulatory SNPs and Their Importance

Following the regulatory SNP (rSNP) detection method of Heinrich et al. [7], we selected the SNPs from the genome data which are located in the promoter regions of *B. napus* genes and analyzed them to detect their impact on the TFBSs. For this purpose, we first extracted the flanking sequence of ±25 bp for each selected SNP resulting in a 51 bp long sequence with the SNP in the central position. Second, we created two copies of the flanking sequence: One with the reference allele in the SNP position, the second with its alternate variant. After that, employing the MATCH™ algorithm [77], both sequences were scanned to predict the TFBSs with their affinity scores. The potential binding affinity of a TF was quantified by MATCH™ in terms of a matrix similarity score (MSS) ∈[0,1], where a MSS value of 1 denotes a complete match in each position of the TFBS. As suggested in [67], we removed all TFBS predictions with MSS values <0.85 and which did not overlap the SNP position in the flanking sequences. Finally, to evaluate the impact of a SNP on the binding affinity of a TF, we inferred four different types of consequences for each SNP-TFBS pair: (i) “No Change”: the SNP has no effect on the TF binding; (ii) “Score-Change”: the binding affinity (i.e., MSS) is changed; (iii) “Loss of TFBS”: a TFBS is only found on the reference allele, while the TFBS does not occur in the alternate allele; and (iv) “Gain of TFBS”: the TFBS appears only for the alternate allele. In the following, we define a SNP as rSNP if it causes a “Gain of TFBS” or a “Loss of TFBS” (consequence iii or iv) for at least one TFBS.

### 3.4. Association Analysis Using Random Forests

For the assessment of the importance of single rSNPs, regarding their association to the *B. napus* cultivars, we applied a random forest (RF)-based feature selection algorithm to measure the relative importance of each rSNP for the trait oil content (congruent with oil quality, see Table 2), following our previous studies [21,22]. In particular, the relative importance of each rSNP is calculated by applying the Boruta algorithm [79], which is an RF-based feature selection wrapper for finding all relevant variables in a data set. The Boruta algorithm assesses important features (in this case rSNPs) with respect to a variable outcome (in this case oil content) by constructing multiple decision trees based on random subsets of attributes or features. The pseudo-code for Boruta is given in Algorithm 1.

Using Algorithm 1, in this study, we analyze genotypes of rSNPs to identify their significant genotype × phenotype association regarding the oil content of the cultivars. In order to deal with remaining obstacles resulting from the correlations between the SNPs or random fluctuations involved in the data set, we iteratively applied the Boruta algorithm (e.g., 1000 times), and considered an rSNP in our further analysis as important if and only if its importance was confirmed in all analyses. In the following, we refer to those rSNPs as *important rSNPs*.
**Algorithm 1** Boruta Algorithm**Input:**M: Genotype (rSNPs) data**Input:**L: Labels (cultivars)**Output:**C: A ranked list of rSNPs based on their importance score**Method:**1:t=02:**repeat**3:    $M_{t}=M$4:    ${\hat{M}}_{t}=\mathrm{shuffle}\left(M_{t}\right)$: Creation of shadow attributes5:    $M_{t}^{ext}=\left[M_{t};{\hat{M}}_{t};L\right]$: Matrix (data) concatenation to extend the input data6:    ${\mathrm{VIS}}_{t}\left(M_{t}^{ext}\right)=RF\left(M_{t}^{ext}\right)$: Gathering variable importance scores (VIS) using RF classifier7:    ${\hat{\mathrm{VIS}}}_{t}=\max\left(\mathrm{VIS}\left({\hat{M}}_{t}\right)\right)$: Max. VIS value (in terms of z-Score) for shadow attributes8:    $M_{t}^{c}=M_{t}^{ext}\left[\mathrm{VIS}\left(M_{t}^{ext}\right)>{\hat{\mathrm{VIS}}}_{t}\right]\backslash {\hat{M}}_{t}$: rSNPs with significantly higher VIS values $>\hat{\mathrm{VIS}}$9:    $M_{t}^{r}=M_{t}^{ext}\left[\mathrm{VIS}\left(M_{t}^{ext}\right)<{\hat{\mathrm{VIS}}}_{t}\right]\backslash {\hat{M}}_{t}$: rSNPs with significantly lower VIS values $<\hat{\mathrm{VIS}}$10:    $M=M_{t}\backslash \left[M_{t}^{c};M_{t}^{r}\right]$: Remove all rSNPs with determined importance from the input $M_{t}$11:    $C_{t}=\mathrm{VIS}\left(M_{t}^{c}\right)$: Gathering the rSNPs with confirmed VIS12:    t=t+113:**until** importance of all rSNPs is assigned14:$C={⋃}_{i=1}^{t}C_{i}$

## 4. Conclusions

Transcription factors orchestrate the entirety of cellular processes leading to tissue development, tissue differentiation or responses to the environment and, hence, act as natural master regulators within plants [39]. This makes them promising candidates as breeding targets to control complex traits in crop breeding [39]. In this study, we performed a systematic analysis using multi-omics data (genomics, transcriptomics, and proteomics) to investigate the complex interplay between rSNPs, TFs and DEGs. As a result of this analysis, we obtained: (i) a genome-wide collection of rSNPs; (ii) their significant association with the *B. napus* cultivars differing in oil content; (iii) their consequences for TF binding; and (iv) the DEGs of four tissues whose expression could be strongly affected by the occurrence of these *important rSNPs* within their promoter regions.

Our findings show that while members of the TF-families DOF, MYB, NAC, GATA, or TCP have been identified as enriched exclusively for a certain tissue, the TFs in the bHLH or bZIP class, and members of the BES1 family seem to play important regulatory roles in several tissues. Moreover, the knowledge on the causal interaction between a rSNP, a TF and a DEG could be promising to explain the expression behavior of the gene, which in turn is essential for understanding the underlying genetic programs such as tissue development or responses to abiotic and biotic stresses.

By mainly considering the promoter regions, our integrated approach provides important insights into the regulatory processes on the transcriptional level. For future work, the investigation of further regulatory mechanisms underlying differential gene expression, as, e.g., post-transcriptional regulation such as microRNA binding or Riboswitch activity can help to gain a comprehensive understanding of the entirety of gene regulatory processes. Nevertheless, our study can be seen as one further step leading towards the deciphering of differential gene expression underlying the different *B. napus* cultivars and our genome-wide collection of rSNPs provides a basis for upcoming studies on different traits in *B. napus*.

## Figures and Tables

**Table 1:** Numbers of differentially expressed genes (DEGs) in four tissues based on the comparison of the cultivars Zhongshuang11 (ZS11) against Zhongyou821 (ZY821). Up-regulated and down-regulated DEGs are defined as log${}_{2}$ fold change >2 and log${}_{2}$ fold change <−2 and an adjusted *p*-value threshold of 0.05, respectively.

Tissue	No. of DEGs	No. of Up-Regulated DEGs	No. of Down-Regulated DEGs
**Flower**	11,442	5221	6221
**Leaf**	3234	1486	1748
**Stem**	4198	2510	1688
**Root**	2318	1448	870

**Table 2:** Meta data of the RNA-Seq experiment samples which were used for differential expression analysis. ZS11 and ZY821 stand for Zhongshuang11 and Zhongyou821, respectively. 00 and ++ stand for low erucic acid, low glucosinolate and high erucic acid, high glucosinolate, respectively.

Cultivar	Oil Quality	Oil Content	Biological Replicates
ZS11	00	high	2
ZY821	++	low	2

## Supplementary Materials

The following are available online at https://www.mdpi.com/1422-0067/22/2/789/s1.
