# Supplementary material for: In Silico Identification of the Complex Interplay between Regulatory SNPs, Transcription Factors, and Their Related Genes in Brassica napus L. Using Multi-Omics Data
Source: Int J Mol Sci. 2021 Jan 14;22(2):789. doi: 10.3390/ijms22020789 (PMC7830561; doi:10.3390/ijms22020789)
Supplement: Supplementary file 1 [file ijms-22-00789-s001.zip › Suppl_Material/Suppl_FiguresS1_treemaps_sig_GO_BP.pdf]

Supplementary Figures S1: Treemaps for the enriched GO terms (biological processes) of the DEGs, separated into up- and down-regulated DEGs

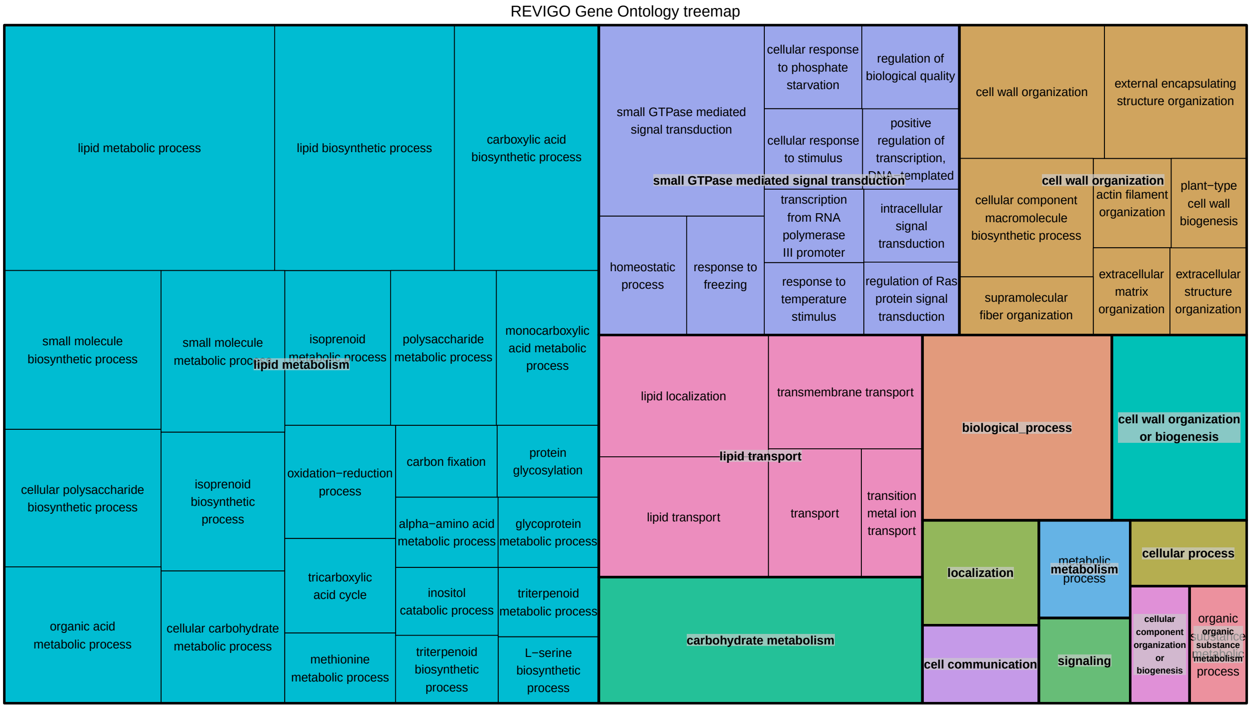

Figure S1.1: Treemap for the enriched GO terms (biological processes) of the up-regulated flower DEGs. The treemap is created using the applications g:Profiler followed by REVIGO.

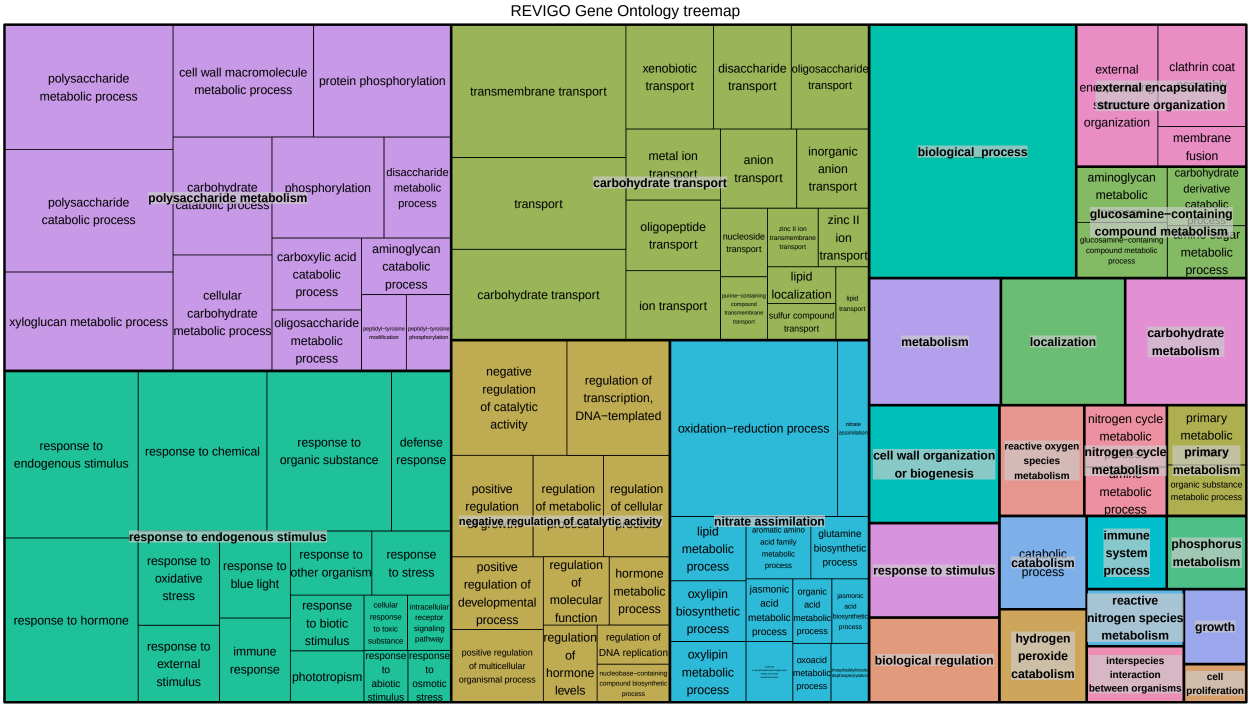

Figure S1.2: Treemap for the enriched GO terms (biological processes) of the down-regulated flower DEGs. The treemap is created using the applications g:Profiler followed by REVIGO.

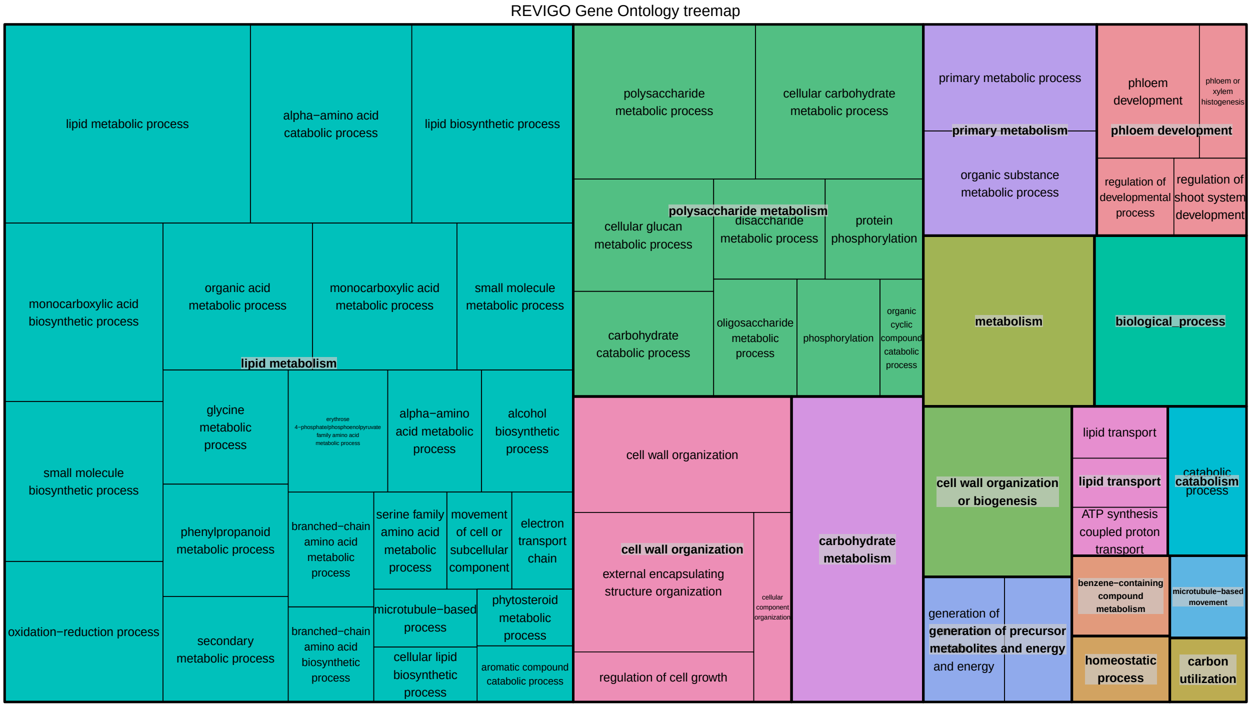

Figure S1.3: Treemap for the enriched GO terms (biological processes) of the up-regulated leaf DEGs. The treemap is created using the applications g:Profiler followed by REVIGO.

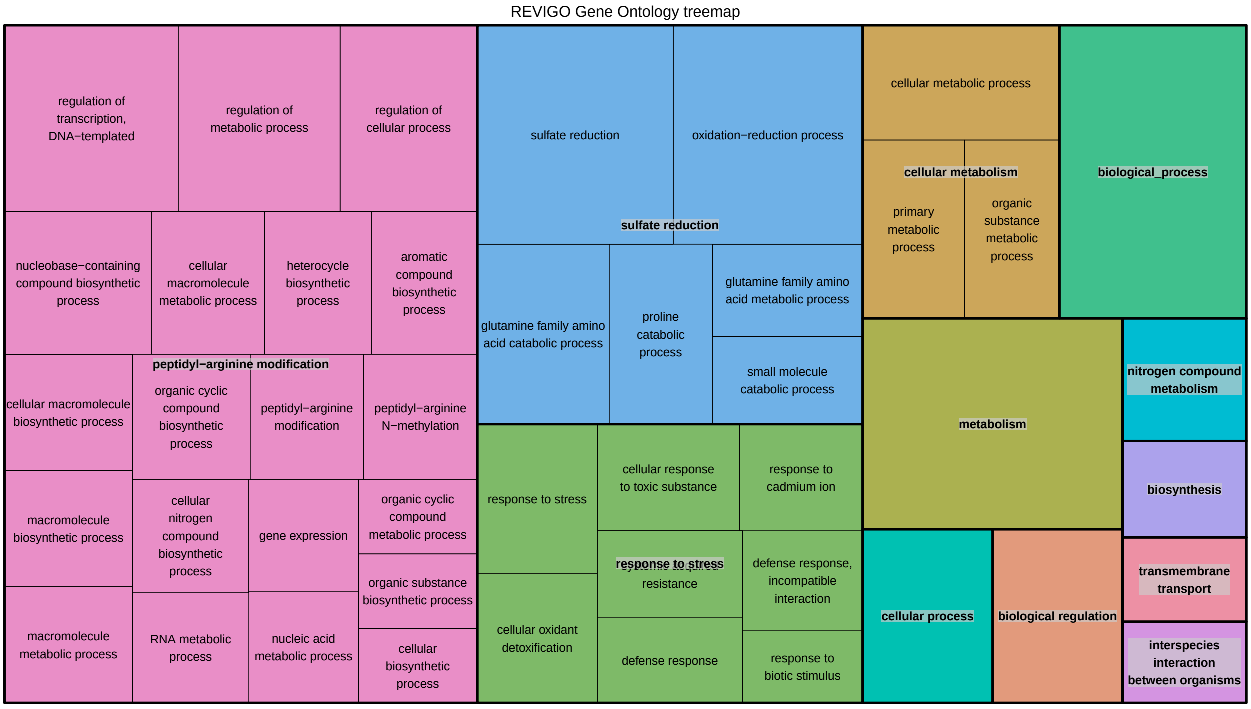

Figure S1.4: Treemap for the enriched GO terms (biological processes) of the down-regulated leaf DEGs. The treemap is created using the applications g:Profiler followed by REVIGO.

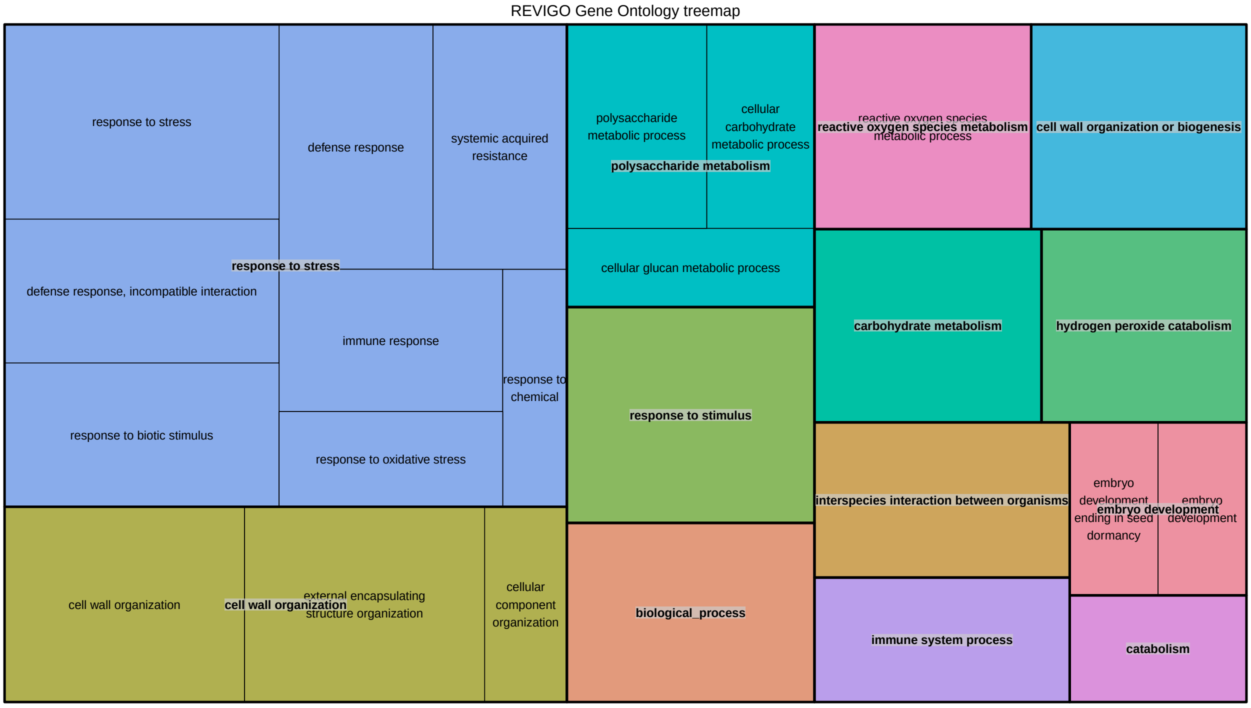

Figure S1.5: Treemap for the enriched GO terms (biological processes) of the up-regulated stem DEGs. The treemap is created using the applications g:Profiler followed by REVIGO.

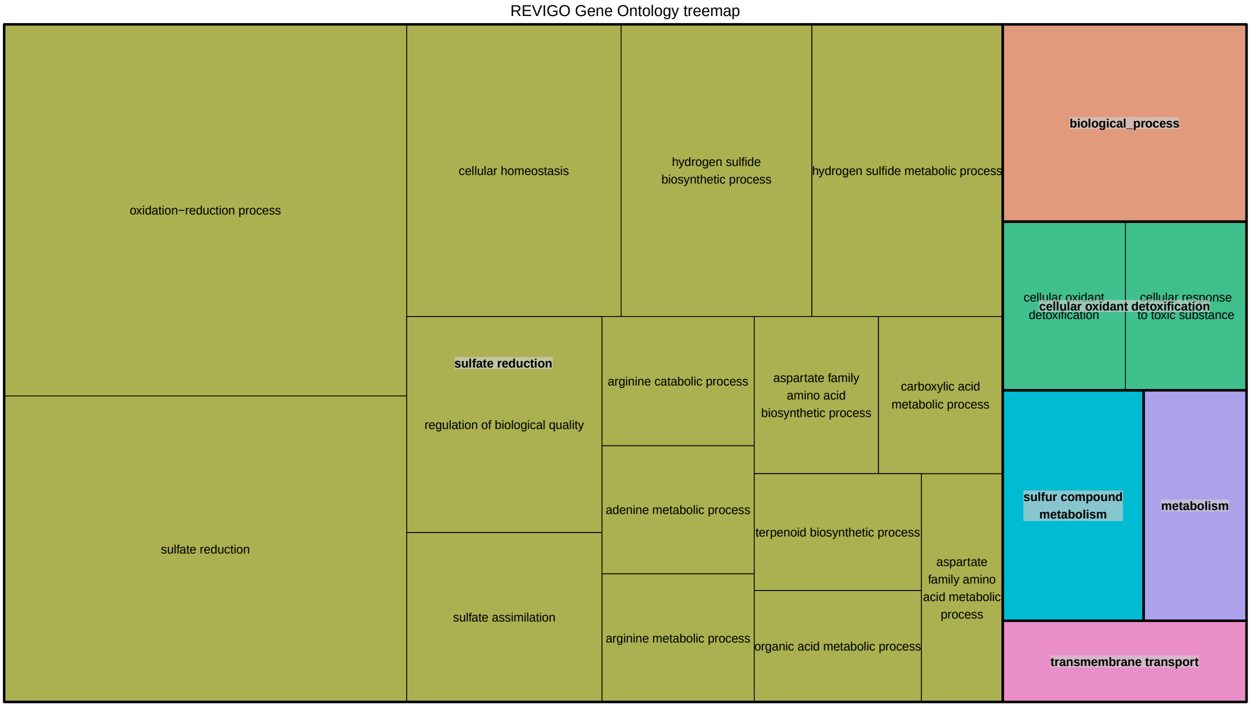

Figure S1.6: Treemap for the enriched GO terms (biological processes) of the down-regulated stem DEGs. The treemap is created using the applications g:Profiler followed by REVIGO.

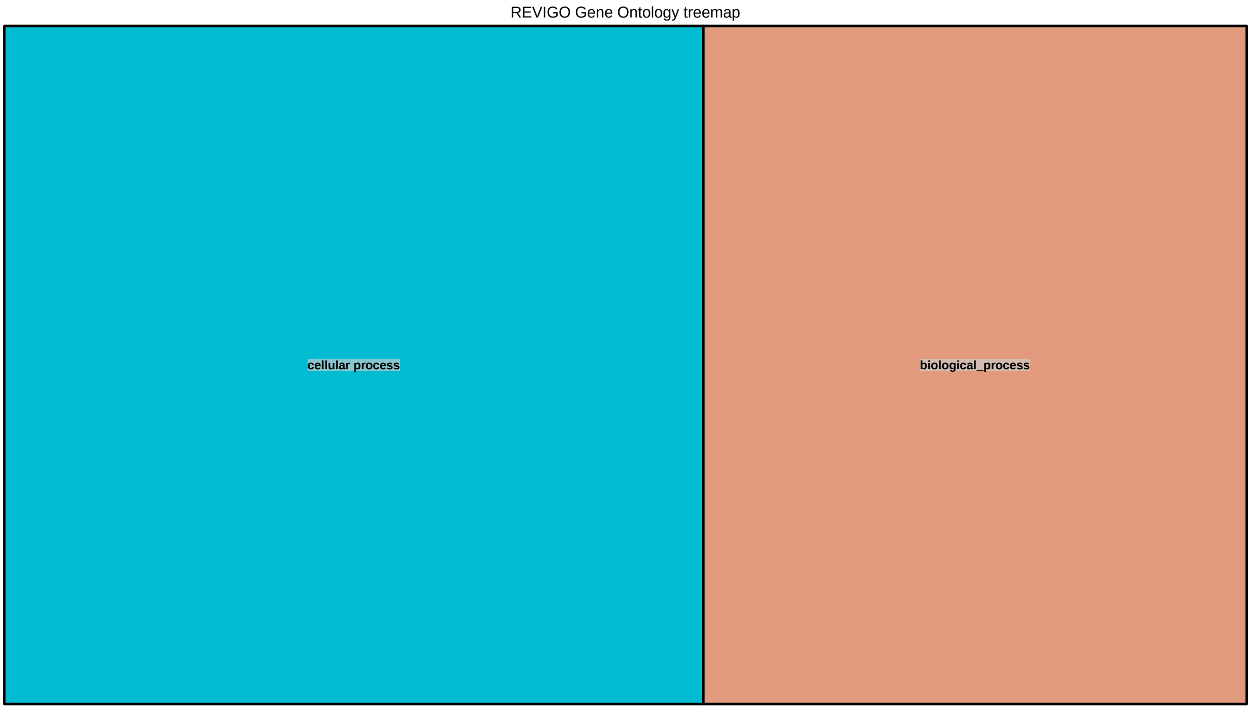

Figure S1.7: Treemap for the enriched GO terms (biological processes) of the up-regulated root DEGs. The treemap is created using the applications g:Profiler followed by REVIGO.

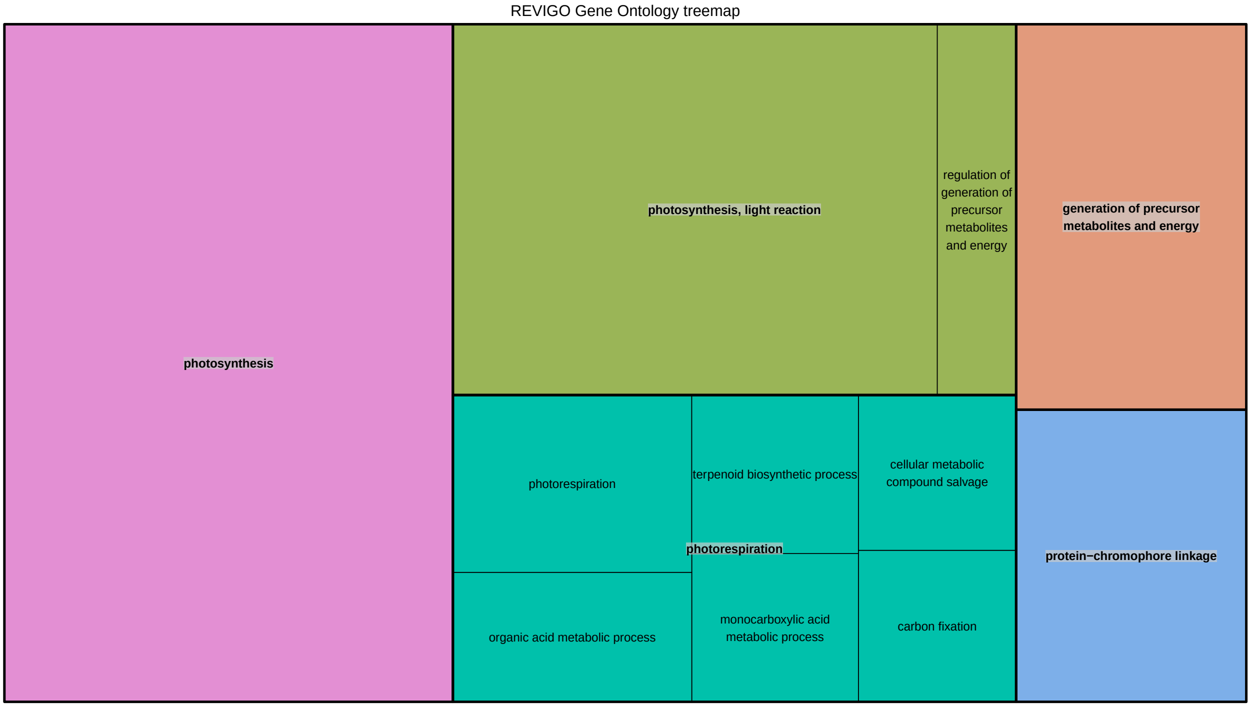

Figure S1.8: Treemap for the enriched GO terms (biological processes) of the down-regulated root DEGs. The treemap is created using the applications g:Profiler followed by REVIGO.
